# Recurrent pneumothorax in a human immunodeficiency virus-positive patient with multidrug-resistant tuberculosis: a rare case of bronchopleural fistula: a case report

**DOI:** 10.1186/s13256-022-03436-1

**Published:** 2022-05-31

**Authors:** Lydia Nakiyingi, Joseph Baruch Baluku, Willy Ssengooba, Sharon Miriam Namiiro, Paul Buyego, Ivan Kimuli, Susan Adakun

**Affiliations:** 1 Department of Medicine, College of Health Sciences, Makerere University, P.O. Box 7072, Kampala, Uganda; 2 Research Department, Infectious Diseases Institute, College of Health Sciences, Makerere University, Kampala, Uganda; 3 Division of Pulmonology, Mulago National Referral Hospital, Kampala, Uganda; 4 Department of Microbiology and Immunology, College of Health Sciences, Makerere University, Kampala, Uganda; 5 Makerere University Lung Institute, Kampala, Uganda

**Keywords:** Multidrug resistant, Tuberculosis, HIV, Bronchopleural, Fistula, Pneumothorax, Case report

## Abstract

**Background:**

Human immunodeficiency virus/tuberculosis coinfections have amplified the multidrug-resistant tuberculosis pandemic in many countries in Sub-Saharan Africa, and multidrug-resistant tuberculosis has become a major public health threat. There is a paucity of data on severe complications of multidrug-resistant tuberculosis in the context of human immunodeficiency virus coinfection despite the increasing prevalence of multidrug-resistant tuberculosis/human immunodeficiency virus coinfection and the complexity of multidrug-resistant tuberculosis treatment. This report describes a rare case of complicated multidrug-resistant tuberculosis in a human immunodeficiency virus-positive individual.

**Case presentation:**

A 39-year-old human immunodeficiency virus-positive Ugandan male on anti-retroviral therapy for 6 years, who had recently completed treatment for drug-susceptible tuberculosis from a public hospital, presented to the tuberculosis ward of Mulago National Referral Hospital with worsening respiratory symptoms including persistent cough with purulent sputum, fever, right chest pain, and shortness of breath. On admission, a diagnosis of drug-resistant tuberculosis was made following a positive sputum Xpert MTB/Rif test with rifampicin resistance. Culture-based tuberculosis tests and line probe assay confirmed multidrug-resistant tuberculosis. The patient was given multidrug-resistant tuberculosis treatment that included bedaquiline, isoniazid, prothionamide, clofazimine, ethambutol, levofloxacin, and pyrazinamide and switched to second-line anti-retroviral therapy that included tenofovir/lamivudine/lopinavir/ritonavir. Chest X-ray revealed a hydro-pneumothorax, following which a chest tube was inserted. With persistent bubbling from the chest tube weeks later and a check chest X-ray that showed increasing pleural airspace (pneumothorax) and appearance of a new air–fluid level, chest computed tomography scan was performed, revealing a bronchopleural fistula in the right hemithorax. The computed tomography scan also revealed a pyo-pneumothorax and lung collapse involving the right middle and lower lobes as well as a thick-walled cavity in the right upper lobe. With the pulmonary complications, particularly the recurrent pneumothorax, bronchopleural fistula, and empyema thoracis, cardiothoracic surgeons were involved, who managed the patient conservatively and maintained the chest tube. The patient continued to be ill with recurrent pneumothorax despite the chest tube, until relatives opted for discharge against medical advice.

**Conclusions:**

Complicated human immunodeficiency virus-related multidrug-resistant tuberculosis is not uncommon in settings of high human immunodeficiency virus/tuberculosis prevalence and is often associated with significant morbidity and mortality. Early diagnosis and treatment of multidrug-resistant tuberculosis, with rigorous monitoring for human immunodeficiency virus-positive individuals, is necessary to prevent debilitating complications.

## Background

Multidrug-resistant tuberculosis (MDR-TB), caused by *Mycobacterium tuberculosis* (*Mtb*) resistant to at least isoniazid and rifampicin, is a growing public health problem globally [1]. HIV/TB coinfections have amplified the MDR-TB pandemic in many countries in Sub-Saharan Africa (SSA). Globally, in 2018, the World Health Organization reported that there were an estimated half a million new cases of rifampicin-resistant TB (of which 78% had MDR-TB) with an estimated 3.4% being new cases and 18% being previously treated cases, and an estimated annual MDR-TB-related mortality of 214,000 deaths [1]. In Africa, the prevalence of drug-resistant TB is estimated to be 2.5% among new cases and 12% among previously treated patients [1, 2].

The emergence of MDR-TB is a threat to populations in SSA due to high prevalence of infectious diseases, especially HIV infection, limited access to well-equipped healthcare facilities in the most resource-poor settings, and the high costs of treatment, thus worsening the effect of MDR-TB [3, 4]. HIV coinfection in MDR-TB has been associated with increased mortality and severe disease complications as a consequence of interconnected challenges related to the complexity of MDR-TB treatment and to clinical management of HIV coinfection [3, 5]. The interconnected challenges that include long duration of treatment, large numbers of daily pills, and drug toxicities, often result in poor treatment outcomes, including mortality [3, 6]. Complications in MDR-TB and HIV coinfection have notably been underreported [3, 6]. The several reports on TB complications in HIV coinfection have mainly been among patients with drug-susceptible TB. However, there is a paucity of data on MDR-TB complications among patients with HIV coinfection in settings with high prevalence of HIV/TB despite the increasing prevalence of MDR-TB/HIV coinfection.

This report describes a rare case of complicated MDR-TB in a HIV-positive individual from a setting with high prevalence of HIV/TB, where a large proportion of MDR-TB patients are also likely to have HIV coinfection. The goal is to highlight to clinicians the possibility of severe complications of MDR-TB that could complicate management of MDR-TB in the face of HIV coinfection.

## Case summary

A 39-year-old Ugandan male was admitted to the tuberculosis (TB) ward of Mulago National Referral Hospital as a referral from a public hospital, for further investigations and treatment following long-standing history of cough and B-symptoms lasting over a period of 1 year despite completion of TB treatment for smear-positive drug-sensitive pulmonary TB (PTB). The patient was first diagnosed with smear-positive PTB at the public hospital, where he was initiated on TB treatment as a new case, with 2 months of isoniazid (H), ethambutol (E), rifampicin (R), and pyrazinamide (Z) for the intensive phase and 4 months of rifampicin(R) and isoniazid (H) for the continuation phase (2HERZ/4RH), according to Uganda National TB Treatment Guidelines [7], which he took to completion. However, despite completion of the recommended 6-month TB treatment regimen, the cough persisted and the patient continued to get B-symptoms, with occasional difficulty in breathing. Following a positive sputum smear at completion of the 6-month TB regimen, the attending clinician at the public hospital made a clinical decision to extend the continuation phase of the TB treatment (RH) for another 2 months, which the patient took without any registered improvement. He was then referred to Mulago National Referral Hospital for further management.

**Fig. 1:**
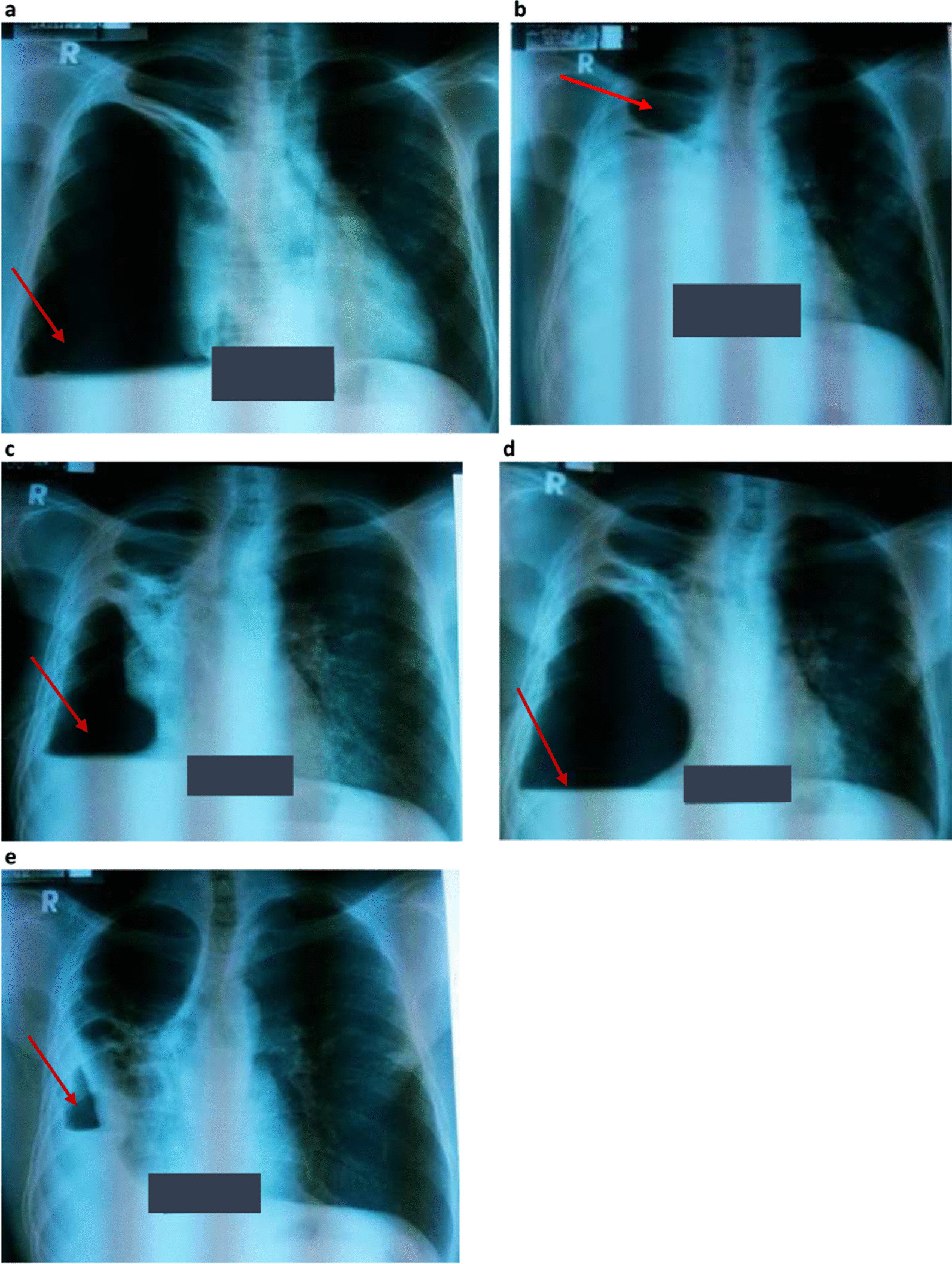
**a**–**e** Selected serial chest X-rays taken at various stages during MRD-TB treatment. **a** Chest X-ray taken when patient first presented to Mulago Referral Hospital tuberculosis ward and was diagnosed with MDR-TB. Chest X-ray shows hydro-pneumothorax (arrow) in the right hemithorax. Chest tube was inserted. **b** Chest X-ray taken a few weeks after the hydro-pneumothorax was managed with underwater seal drainage (chest tube). Chest X-ray shows a cavity (arrow) in the upper zone of the right hemithorax following removal of the chest tube. **c** Chest X-ray taken 2 months post MDR-TB treatment, when the patient reported new symptoms of cough, chest pain, and shortness of breath. Chest X-ray still showed hydro-pneumothorax (arrow) in the right hemithorax. Chest tube was reinserted. **d** Chest X-ray showing increasing pneumothorax (arrow) and a new air–fluid level in the right hemithorax despite a chest tube *in situ*. Chest computed tomography scan was performed at the time. **e** Chest X-ray showing recurring pneumothorax (arrow) with a new air–fluid level in the right hemithorax at 2 months following bronchopleural fistula diagnosis

**Fig. 2:**
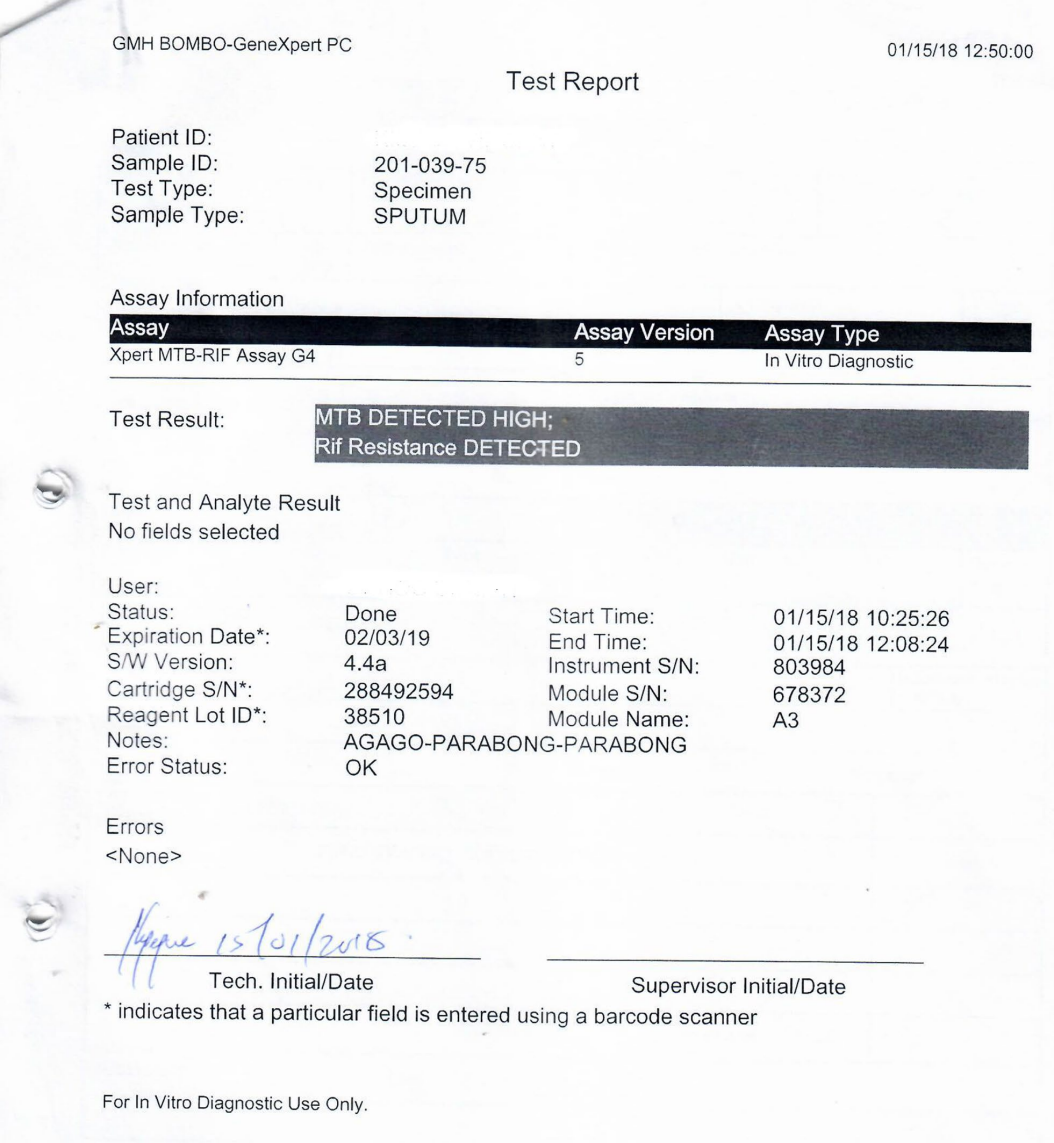
GeneXpert laboratory report showing microbiological evidence of Multidrug-resistant tuberculosis on a spot sputum sample collected on the patient’s first visit to the referral hospital

At the initial visit to the TB clinic of Mulago Hospital, the patient reported a persistent productive cough with yellow sputum and B-symptoms despite taking first-line TB treatment for almost 8 months. He also reported general malaise, right chest pain, and shortness of breath, but no wheezing. Physical examination revealed a sick young male, wasted (body weight 44 kg with body mass index (BMI) of 15 kg/m^2^). His body temperature was 36.7 °C, and he had mild pallor of mucus membranes but without lymphadenopathy or pedal edema. He had tachycardia of 123 beats/minute with normal blood pressure of 110/70 mmHg. Respiratory examination revealed features suggestive of cavitation (amphoric breathing on auscultation) and a pneumothorax (hyperresonant percussion note with absent breath sounds) on the right hemithorax, which was further confirmed on chest X-ray (CXR) (Fig. 1a). Auscultation of the precordium revealed normal heart findings. An Xpert MTB/RIF test performed on a spot sample was positive for *Mycobacterium tuberculosis* (*Mtb*) with rifampicin resistance (Fig. 2), and smear microscopy on fluorescence microscopy on the same sample was also positive for acid-fast bacilli (AFB) grade 2+. Following these tests, a diagnosis of MDR-TB complicated by right pneumothorax was made, with a plan to initiate the patient on MDR-TB treatment following pretreatment assessment and investigations and to immediately relieve the pneumothorax by insertion of a chest tube (also known as underwater seal drainage). The chest tube was immediately inserted to relieve the pneumothorax in the right hemithorax, which significantly reduced, as shown on the check CXR following chest tube removal (Fig. 1b). An early-morning sample taken a day following admission revealed positive Mycobacterial Growth Indicator Tube (MGIT) and Lowenstein–Jensen (LJ) cultures for MTB with confluent growth, although the TB culture results were received several days after the initial confirmation of the diagnosis of MDR-TB on Xpert MTB/RIF test. According to the TB guidelines, presence of rifampicin resistance on a positive Xpert MTB/RIF test is indicative of MDR-TB. Line probe assay, HAIN GenoType MTBDR*plus*, and HAIN GenoType MTBDR*sl* further confirmed *Mtb* complex with resistance to rifampicin and isoniazid but susceptibility to second-line TB drugs, i.e, fluoroquinolones (FLQ) (ofloxacin and moxifloxacin) and injectables (kanamycin, amikacin/capreomycin, and viomycin).

All routine pre-MDR-TB treatment investigations were normal except for mild normocytic normochromic anemia (hemoglobin level 10.2 g/l) and sinus tachycardia with mild right axis deviation on electrocardiogram. Viral hepatitis B and C screening was performed as part of pre-MDR-TB treatment preparation and found to be negative. Renal function tests, liver function tests, serum electrolytes, urinalysis, audiometry, visual, and color tests were all normal. The CD4 cell count was 625 cells/µL, and HIV viral load was undetectable on RNA polymerase chain reaction (PCR).

Regarding MDR-TB treatment, the patient was enrolled into an ongoing clinical trial on the TB ward of Mulago Hospital, in which he was initiated on a bedaquiline-based regimen which is superior when compared with standard of care as described in the Uganda National TB diagnosis and treatment guidelines [7] at the time. The MDR-TB treatment regimen that the patient was given included bedaquiline, high-dose isoniazid, prothionamide, clofazimine, ethambutol, levofloxacin, and pyrazinamide. In addition, the patient was given pyridoxine tablets to minimize isoniazid side effects, particularly isoniazid-induced neuropathy. Due to potential undesired efavirenz–bedaquiline drug interactions, the patient’s anti-retroviral therapy (ART) regimen was switched from tenofovir/lamivudine/efavirenz, which he had taken for 6 years, to a second-line ART regimen including tenofovir/lamivudine/lopinavir/ritonavir. The second-line ART regimen was believed to be better tolerable and was permissible for the TB trial. The patient also continued to take regular cotrimoxazole prophylaxis for opportunistic HIV infections. Serial CD4 cell counts and viral load tests performed as part of the patient’s HIV treatment monitoring indicated that he had sustained viral suppression, and his CD4 cell count ranged between 600 and 700 cells/µL. A month later, there was improvement in the pneumothorax with evidence of lung expansion on CXR (not shown).

Two months following MDR-TB treatment that was taken regularly under directly observed therapy (DOT), the patient still reported persistent cough with purulent sputum, and on and off low-grade fevers, although the excessive sweats and anorexia had subsided. The patient also reported persistent right chest pain, easy fatigability, and difficulty in breathing but no wheezing. On physical examination, the patient was dehydrated, and dyspneic with a respiratory rate of 27 breaths/minute and 91% oxygen saturation. He was febrile (*T* = 37.8 °C) and wasted (body weight 45 kg, BMI 15.5 kg/m^2^). Repeat CXR at this time (Fig. 1c) revealed a large cavity in the upper right lung field with features of a new hydro-pneumothorax on the same right hemithorax. There were no features to suggest tension pneumothorax on either physical examination or CXR. Aspiration of pleural fluid revealed empyema thoracis. The patient was thus diagnosed with recurrent pneumothorax and empyema thoracis in the right hemithorax.

**Fig. 3:**
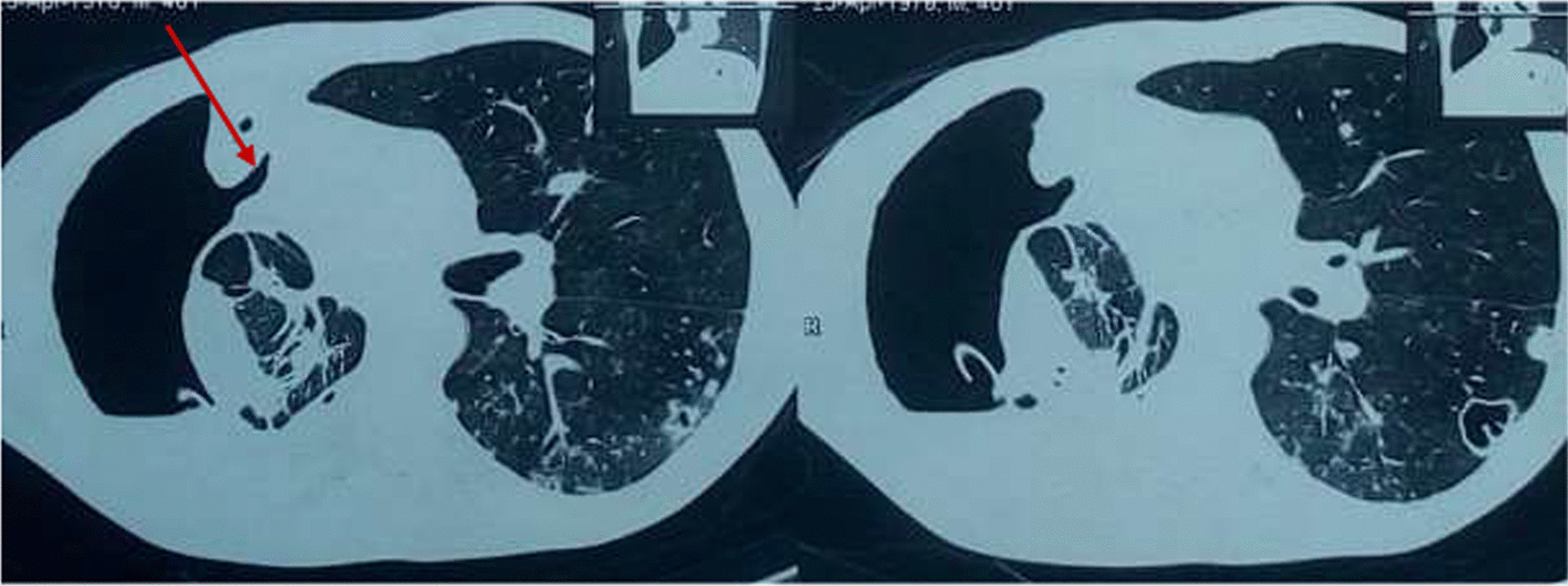
Computed tomography scan segments showing bronchopleural details. Computed tomography scan segments showing details of bronchopleural fistula in the right hemithorax (arrow), pyo-pneumothorax, partial right middle and lower lung lobe collapse, and thick-walled cavity in the right upper lobe of the right hemithorax

As part of his management plan at the time, a cardiothoracic surgery team was immediately contacted for urgent assessment and management of the patient, and the surgeon promptly reinserted a chest tube. In addition, the patient was initiated on antibiotics, which included intravenous ceftriaxone and oral metronidazole for a period of 2 weeks, followed by a week of oral antibiotics with metronidazole and oral azithromycin. The patient reported slight improvement in symptoms, particularly difficulty in breathing and right chest pain, in the first few days after insertion of the chest tube. However, on daily monitoring of the chest tube drainage, persistent bubbling was noted after almost a week of insertion. A check CXR (Fig. 1d) was performed, which showed worsening pneumothorax and appearance of a new air–fluid level in the right hemithorax, despite presence of the chest tube. This was indicative of a possible communication between the bronchial tree and the pleural space. With the findings of persistent bubbling on chest tube drainage monitoring and increasing pneumothorax on check CXR, a chest computed tomography (CT) scan (Fig. 3) was performed, revealing a bronchopleural fistula in the right hemithorax. In addition, the chest CT scan showed a large pyo-pneumothorax with partial right middle and lower lung lobe collapse, a large thick-walled cavity in the residual right upper lobe, and mild mediastinal shift to the right side.

A diagnosis of an HIV-positive patient with MDR-TB complicated with a bronchopleural fistula and pyo-pneumothorax was made. The management for the pulmonary complications (bronchopleural fistula and pyo-pneumothorax) was taken over by the cardiothoracic surgeons, who maintained the chest tube drainage. The patient continued his MDR-TB treatment and ART as prescribed and was monitored for any complications that could arise from presence of a bronchopleural fistula, especially tension pneumothorax. The patient continued to be ill with respiratory symptoms, right chest pain, and occasional difficulty in breathing. Although a repeat CXR had indicated improvement in the pneumothorax after diagnosis of the bronchopleural fistula and management with a chest tube, a CXR (Fig. 1e) performed a month later showed recurring pneumothorax.

Concerning MDR-TB treatment, monthly monitoring of sputum with TB cultures indicated presence of culture conversion at approximately 4 months post MDR-TB treatment initiation. While on the ward, the patient continued to develop recurrent pneumothorax, possibly resulting from the bronchopleural fistula, despite the management he was receiving, until the family opted for his discharge from the ward against medical advice.

Notably, in the family and social history, it was discovered that the patient was in a HIV-discordant relationship (his wife being HIV negative) and had four children who lived with him in a small, one-roomed house. The patient reported taking moderate alcohol, mainly a local gin called *waragi*, until 2 months prior to the current readmission, when he stopped because he believed that the persistent cough was resulting from his social habits. The household contacts were traced and screened for TB as well. None of the contacts were found to have active TB. The patient received counseling about his social habits and possible ways to avoid transmission of MDR-TB to his contacts, especially his immediate family.

## Discussion

This case highlights a rare event of bronchopleural fistula, possibly resulting in recurrent pneumothorax that complicated MDR-TB treatment in a patient with HIV coinfection. This case was further complicated by empyema thoracis (pyogenic pleural effusion), a condition that often causes severe illness and severe sepsis. These multiple complications in HIV coinfection affect MDR-TB and can frustrate the clinician and patient, often resulting in poor treatment outcome. As described in this report, the patient continued to be ill with recurrent pneumothorax despite treatment, which could also explain why the patient and his family opted for discharge from the hospital, against medical advice. This case illustrates the difficulties that may be encountered in the management of MDR-TB in settings with high prevalence of HIV/TB, where a large proportion of MDR-TB patients are also likely to have HIV coinfection. Management of MDR-TB/HIV coinfection is even more challenging when MDR-TB is associated with severe complications, as seen in our case. Our case further illustrates the need for a multidisciplinary approach in the management of MDR-TB in settings with high prevalence of HIV and TB. MDR-TB management in our case required involvement of cardiothoracic surgeons, radiologists, TB clinicians, and HIV physicians. The multiple medical and surgical disciplines may not be readily available or accessible in most healthcare facilities in resource-poor settings, which worsens the effect of MDR-TB.

HIV/TB coinfections have amplified the MDR-TB pandemic in many countries in Sub-Saharan Africa, leading to several additional HIV/TB treatment challenges [1–6]. Successful MDR-TB treatment presently requires prolonged administration of multiple, poorly tolerated antimicrobial drugs to eradicate active infection. Optimal treatment of MDR-TB has historically required the use of five to seven anti-mycobacterial drugs for 24 months, including an injectable agent such as an kanamycin or capreomycin for at least the first 6 months. The recent World Health Organization (WHO)-recommended short-course, modified Bangladesh regimen that has been rolled out in several settings retains the need for administration of toxic injectable agents daily for at least the first 6 months of treatment [1, 7] and the likelihood of treatment success declines as resistance accumulates [1]. The said patient who was initiated on seven [7] antimicrobial drugs for MDR-TB (bedaquiline, isoniazid, prothionamide, clofazimine, ethambutol, levofloxacin, and pyrazinamide) and pyridoxine also had to endure multiple antiretroviral therapy (ART) drugs and cotrimoxazole prophylaxis for HIV coinfection. In addition to issues that could be related to the high pill burden and shared drug side effects, clinicians needed to watch out for drug–drug interactions between the said drugs, which could affect the efficacy of the regimens. For this reason, the patient’s ART regimen was switched to what was thought to be a more tolerable regimen.

In addition to the multiple drug issues, the patient also developed a bronchopleural fistula, which could have resulted in recurrent pneumothorax and made the patient very sick. A bronchopleural fistula is a communication between the pleural space and the bronchial tree [8, 9]. Although rare, bronchopleural fistulae represent a challenging management problem and are associated with high morbidity and mortality [10]. The most common cause of bronchopleural fistula is as a complication following pulmonary resection. Other causes are lung necrosis complicating infection, persistent spontaneous pneumothorax, chemotherapy or radiotherapy (for lung cancer), and TB [8–11]. Our patient had not undergone any pulmonary resection surgery, thus the bronchopleural fistula may have resulted from the rarer causes, particularly lung necrosis as a complication of an infection, in this case TB. The other possibility in our patient is that the bronchopleural fistula could have developed as a complication of persistent spontaneous pneumothorax. It is also possible that the bronchopleural fistula in our patient resulted from a posttraumatic event. However, our patient did not report history to suggest any traumatic event involving the chest.

The diagnosis and management of bronchopleural fistula remain a major challenge for clinicians [10], and localization of bronchopleural fistula may require multiple imaging and bronchoscopies [12]. Successful management of a fistula is combined with treatment of the associated empyema cavity. The first step, therefore, should be control of active infection and adequate drainage of the hemithorax [10]. The diagnosis of bronchopleural fistula in our patient was only by chest CT scan because bronchoscopy could not be performed for a patient who had active MDR-TB infection. Localization of the bronchopleural fistula in this patient therefore remained challenging. The patient could probably have benefited from definitive surgical repair of the fistula [12]. However, without thorough investigations and localization of the fistula, surgery could not be considered; moreover, the capacity to perform such surgeries in this low-income setting was very limited. Further, the patient had active MDR-TB disease, which could have put the surgeons at risk of MDR-TB acquisition. On the other hand, bronchoscopic methods [12, 13] that have been used to close the fistula could probably have been considered in this patient had it not been for active MDR-TB or limited investigations and intervention resources. Our patient’s complications were therefore managed conservatively by the cardiothoracic surgeons with just chest tube drainage. The intention of the chest tube was to drain the associated empyema and the pneumothorax. In addition, antibiotics were empirically given to treat suspected superimposed bacterial infection.

Despite sputum TB culture conversion (negative TB cultures) at 4 months after initiation of MDR-TB treatment, the patient continued to be very ill with recurrent pneumothorax, possibly resulting from the bronchopleural fistula and its complications. This observation further confirms the existing literature on bronchopleural fistula as a serious treatment challenge and significant factor for morbidity and mortality in most patients [10].

In summary, we describe a rare case of MDR-TB in an HIV-positive patient who developed several pulmonary complications including empyema thoracis, recurrent pneumothorax, and bronchopleural fistula. Management of the patient’s complications required a multidisciplinary approach including TB and HIV clinicians, radiologists as well as cardiothoracic surgeons, although proper management was limited by lack of resources. In addition to the pulmonary complications, this MDR-TB patient with HIV coinfection had to endure a high pill burden including a multiple antimicrobial MDR-TB drug regimen, ART regimens, antibiotics for empyema, as well as cotrimoxazole prophylaxis for opportunistic HIV infections.

## Conclusion and recommendation

Complicated MDR-TB is not uncommon in HIV coinfected patients in settings with high prevalence of HIV/TB and is often associated with significant morbidity and mortality. Early diagnosis and treatment of MDR-TB with rigorous monitoring for HIV-positive individuals is necessary to prevent debilitating complications.

## Data Availability

All data generated during this report are included in this published article.

## Abbreviations

ART: Antiretroviral therapy
CT: Computed tomography
CXR: Chest X-ray
LJ: Lowenstein–Jensen
MDR-TB: Multidrug-resistant tuberculosis
MGIT: Mycobacterial growth indicator tube
*Mtb*: *Mycobacterium tuberculosis*
Pulmonary tuberculosis: Tuberculosis
SSA: Sub-Saharan Africa
TB: Tuberculosis
